# Intensive medical student involvement in short-term surgical trips provides safe and effective patient care: a case review

**DOI:** 10.1186/1756-0500-4-317

**Published:** 2011-09-01

**Authors:** Ira L Leeds, Francis X Creighton, Matthew A Wheatley, Jana B Macleod, Jahnavi Srinivasan, Marie P Chery, Viraj A Master

**Affiliations:** 1 Emory University School of Medicine, 1648 Pierce Drive, Atlanta, GA 30322 USA; 2 Department of Emergency Medicine, Emory University School of Medicine, 1648 Pierce Drive, Atlanta, GA 30322 USA; 3 Department of Surgery, Aga Khan University Hospital, 3td Parkland Ave, PO Box 30270-00100 GPO, Nairobi, Kenya; 4 Department of Surgery, Emory University School of Medicine, 1648 Pierce Drive, Atlanta, GA 30322 USA; 5 Project Medishare for Haiti, Rue Tamarin #9, Thomonde, Haiti; 6 Department of Urology and Winship Cancer Institute, Emory University School of Medicine, 1648 Pierce Drive, Atlanta, GA 30322 USA

## Abstract

**Background:**

The hierarchical nature of medical education has been thought necessary for the safe care of patients. In this setting, medical students in particular have limited opportunities for experiential learning. We report on a student-faculty collaboration that has successfully operated an annual, short-term surgical intervention in Haiti for the last three years. Medical students were responsible for logistics and were overseen by faculty members for patient care. Substantial planning with local partners ensured that trip activities supplemented existing surgical services. A case review was performed hypothesizing that such trips could provide effective surgical care while also providing a suitable educational experience.

**Findings:**

Over three week-long trips, 64 cases were performed without any reported complications, and no immediate perioperative morbidity or mortality. A plurality of cases were complex urological procedures that required surgical skills that were locally unavailable (43%). Surgical productivity was twice that of comparable peer institutions in the region. Student roles in patient care were greatly expanded in comparison to those at U.S. academic medical centers and appropriate supervision was maintained.

**Discussion:**

This demonstration project suggests that a properly designed surgical trip model can effectively balance the surgical needs of the community with an opportunity to expose young trainees to a clinical and cross-cultural experience rarely provided at this early stage of medical education. Few formalized programs currently exist although the experience above suggests the rewarding potential for broad-based adoption.

## Background

Historically, the hierarchical nature of medical education with step-wise progression to greater abilities and responsibilities has been thought necessary for the safe and efficient practice of medical care within the academic learning environment. Although such a system has advantages, one cost of this well-established hierarchy is that experiential learning only minimally reaches those further down the chain of command. Medical students in particular have limited clinical responsibilities and therefore limited opportunities for direct hands-on learning considered by some to be the superior form of medical education [1].

In the surgical specialties, this problem is magnified by the nature of procedural medicine. Prior studies[2,3] have demonstrated multiple systemic barriers to surgical skill acquisition by medical students. Given the intrinsic importance of technical acumen and repetition-based learning for surgery,[4] any hierarchy that limits practice of clinical skills may further distance the medical student from the proven ideal form of surgical skill acquisition - routine reiteration [5,6].

One could argue that the rate-limiting step for education of the medical student is not the student, but a system that is overloaded with too many intermediaries. Are medical students unable to work more independently or is this hierarchical construct more a matter of practicality than absolutely necessary supervision? Such inquiry applies not only to student participation in surgical procedures but also students' role in pre- and postoperative patient care.

We report here on a student-faculty collaboration that has successfully operated an annual, short-term surgical intervention in Haiti for the last three years. Medical students were responsible for almost all logistical arrangements and were directly overseen by faculty members for all aspects of patient care, without an intervening layer of resident supervision. It has previously been argued[7,8] that short-term surgical trips can be of potential benefit to underserved populations even with their recognized limitations[9,10]. A less well-explored matter is how and if medical student education should be integrated into such humanitarian missions. We obtained clinical data from these surgical trips to Haiti to explore whether this collaborative model can safely supplement local surgical services in under-resourced areas while also substantively serving as a unique educational opportunity for medical students.

## Methods

From 2001 to the present, a partnership between multiple U.S. academic medical centers and Project Medishare for Haiti has held an ongoing series of student-faculty collaborative health projects in the rural communities of Haiti's *Plateau Central*. In 2007, Emory Medishare, an affiliate based out of Emory University, began operating an annual surgical trip to help supplement services available at *Hôpital St. Thérèse*, the province's main tertiary care hospital located in Hinche, the plateau's provincial capital and largest city. The population of the *Centre *province is over 560,000 [11]. Although the hospital facility has the capacity to accommodate 4-5 surgeons in full-time practice, the remoteness of this region of the country and poor financial incentives limit the human resources available. The hospital typically employs one surgeon on its permanent staff. These local surgeons are also expected to fill full-time administrative posts in the hospital that often prevent them from performing elective procedures on a timely basis. The dearth of surgical providers in the area was a major catalyzing factor for the rapid deployment of Emory Medishare's short-term surgical trips.

### Project Structure

Multiple pre-trip needs assessments were conducted by a multidisciplinary physician team, including, importantly, an in-person survey of the hospital's surgical facilities by one of the attending surgical staff. These investigations helped provide realistic expectations for locally available equipment and supplies as well as establishing long-term local partnerships. An explicit purpose of these exploratory components was to engage with the local surgical team to minimize overlap and maximize the availability of surgical care in the region. Efforts were made to involve local trainees at various Haitian educational institutions as well but strictly structured curricula prevented cross-institutional collaboration.

With the initial clinical care goals met, an evolution occurred across multiple trips to give increasing amounts of responsibility to the medical students. By the third year of the surgical trip, nearly all trip planning, fundraising, physician and staff recruitment, and medical supply and pharmaceutical procurement was performed by a team of ten medical students. Average total trip costs were approximately $24,000 with transportation costs 2/3 of the total (Leeds, unpublished data) which compares favorably with previously reported costs of mission [12].

A specific focus of these efforts was to ensure that all local supply stocks were replenished by items brought by the surgical team. Partners in Health/*Zanmi Lasante*, a partner NGO of Project Medishare who maintains a number of surgical hospitals across Haiti, was instrumental in providing otherwise undeliverable bulk items such as intravenous fluids and oxygen tanks. Project Medishare's local staff helped facilitate logistical services in Haiti (e.g., transportation, room and board). Faculty physicians served as technical advisors ensuring procurement needs matched the needs of the expected surgical cases and provided appropriate legal supervision as required (e.g., receipt and export of controlled pharmaceutical products). Interpretive services were provided by a combination of trained medical interpreters, local English-speaking staff, and Kreyol-speaking, American mid-level providers.

### Clinical Setting

Local community health workers, radio announcements, and direct physician referrals were used in the weeks leading up to each trip to notify the local population of the availability of surgical care for problems that may been previously diagnosed as inoperable. Medical students worked in teams of two or three with an effort to group students to complement each other's knowledge and clinical skills. Patients from Hinche and surrounding rural areas were evaluated initially by these medical student teams and then presented to faculty surgeons in the traditional manner at many academic medical centers, with particular regard to their preoperative fitness for surgery, and the amount of postoperative care required. Medical students then produced - with faculty oversight - a final assessment and plan for each patient. Potential cases were frequently discussed with local surgeons to optimize case selection to maximize clinical effectiveness and productivity of the visiting team. All major cases were undertaken only with the two attending surgeons in agreement with the proposed management plan. Common reasons for declining a surgical referral were diagnostic uncertainty with unclear surgical benefit and poorly controlled medical comorbidities. When possible, these patients were referred to *Hopital de l' Universite d'Etat d'Haiti *in Port-au-Prince for further care.

Patients underwent a variety of surgical procedures with a preference, given the skill sets of the attending surgeons, for complex urological and general surgical cases that required skills and knowledge that were not routinely available in Hinche. Surgeons also balanced conservative case selection due to limited intensive care resources with an urgency to perform procedures that were or could become life threatening if deferred. Academic faculty supervised all patient care. Visiting and local surgical team members often worked side-by-side in both the operating room and inpatient ward. All cases were conducted over a period of four days each year. All appropriate time outs, checks for sterile instruments, and preoperative antibiotic administrations were performed with each case. A particular focus was that medical students should be encouraged to make all point-of-care decisions while being carefully supervised by faculty and staff. Expectations set during first day orientation with both local and visiting teams demanded that quantity and quality of supervision exceed that of a U.S. teaching hospital. All post-operative care was performed by medical students overseen by attending physicians and supplemented by highly experienced critical care nursing staff.

After 48 hours of post-operative management by the surgical team, patients were evaluated for disposition with options including extended postoperative care, assignment to general nursing care provided by the hospital, transfer to another service (e.g., internal medicine for persistent fever), or discharge to home. By design, surgical cases that would routinely require extensive post-operative care were avoided, and cases that had a greater likelihood of longer inpatient care were scheduled earlier in the operative week. Non-operating days were included each year as well to address any unexpected post-operative outcomes. Any patients still under the care of the surgical team at the end of each trip were transferred to the direct care of local surgeons and the community health workers of Project Medishare after an in-person, bedside transfer of care. These local partners were the same providers who actively worked alongside the visiting surgical team for the prior week.

### Participants

Table 1 demonstrates the typical make-up of the surgical team. This group of individuals was responsible for all direct patient care from admission to discharge. In 2010, the visiting surgical team was composed of the following: one attending urologist, one attending general surgeon, one attending anesthesiologist, two anesthetists, one surgical physician's assistant, three critical care registered nurses, an American-born Kreyol interpreter, six third-year medical students, and four second-year medical students. Four members of the team spoke Hatian Kreyol and often served as interpreters. Team composition for 2008 and 2009 was similar with slightly more emphasis on Kreyol language proficiency. All visiting team members were also provided safety and emergency preparedness training. The authors' home institution's travel and evacuation insurance automatically covered the team's needs in Haiti, and Project Medishare's general liability insurance covered all medical liability.

**Table 1:** Make-up of surgical team

	**2008**	**2009**	**2010**
	
**Visiting Team**			
Attending surgeons	2	2	2
Attending anesthesiologist	0	1	1
Other attending-level clinician	2	1	0
Mid-level anesthesia personnel	2	2	2
Registered nurses with Kreyol language skills	4	4	4
Scrub technicians with Kreyol language skills	2	2	1
"Cultural agents" (interpretation and logistics)	0	0	1
Medical students	10	10	10
**Local collaborators**			
Attending surgeons	2	2	1
Attending anesthesiologist	1	1	1
"Cultural agents"	2	2	2
OR Services Staff (room turnover, sterilization)	4	4	4

All third-year medical students participated as part of a scheduled elective week during their surgical clerkship with all the required evaluations and clinical training performed by the two attending surgeons on the trip. Efforts to maintain parity between student learning on this nontraditional elective and the home institution's curriculum has been previously described [13]. Students were selected by open application from the authors' home institution. In general, the vast majority of students had minimal prior experience working abroad in a healthcare setting and brought no unique skills that were beyond the typical qualities of an early clinical trainee. All medical students received additional pre-trip teaching sessions to reinforce critical concepts for intensive overseas surgical care. The topics of these sessions included advanced suturing techniques, preoperative medical assessment, postoperative anesthesia care, and basic bedside nursing care. All students were also introduced to the ethical framework employed by Emory Medishare developed by Suchdev *et al *specifically for short-term medical missions [14].

In addition to the team brought from Emory University, the trip was joined by a number of local team members as well. The hospital director of Hôpital St. Thérèse and an attending surgeon, a registered nurse from Project Medishare, two charge nurses, and many other operating room and ward staff were instrumental in helping the visiting surgical team work within the government-funded Haitian health system.

### Study Design

A review of clinical case records was performed from the three surgical trips spanning July 2008 through July 2010. These items were pulled directly from Emory Medishare medical records that had been stripped of any nonpertinent patient-identifying information. Data collected included clinical productivity data (e.g., operating room time, case burden, etc.), complications or reoperations, and the roles performed by medical students. This clinical data was deemed to be exempt from full review by the Emory University Institutional Review board and permission was given by Emory Medishare to publish these results. Data collection was also approved by Project Medishare and by local representatives of the Haitian Ministry of Health.

Short-term complications were tracked for the first forty-eight hours after the patient left the care of the perioperative anesthesia providers. Reportable events included uncontrollable bleeding, symptomatic hypovolemia, hypoxemia, peritoneal signs, sepsis, return to the operating room, uncontrollable pain, uncontrollable fever, and death. Long-term complications were tracked by the surgical team's local collaborators. Project Medishare's medical director and the hospital director were both charged with reporting any long-term complications for up to one year following a patient's procedure. Reportable events included but were not limited to wound infection, wound dehiscence, hospital readmission, failure of disease or symptom resolution, and death.

## Findings

### Population Characteristics

Over the three year period, a total of 64 procedures were performed on 54 patients in 10 operative days. Forty-seven of these patients were male and 7 were female.

Comprehensive patient demographic data was only available for July 2010. Of patients that were taken to surgery, the average age of patients was 38 years old (median age = 44 years old) with a range of 5 years old to 77 years old. Although records were not available, the average age of patients has likely trended downward from 2008 to 2010 as the surgical team performed more pediatric procedures (e.g., orchiopexy for cryptorchidism) and less prostate-related procedures (e.g., simple prostatectomy for benign prostate hyperplasia(BPH)). Most patients came from Haiti's local *Plateau Central *region but some patients reported - across multiple years - traveling from northern Haiti and Port-au-Prince for procedures that were too costly or unavailable.

### Case Load

Table 2 demonstrates a year-by-year comparison of surgical cases performed on each trip reported here. In 2008, a total of 15 procedures (14 male, 1 female) were performed with an estimated 30 patients evaluated in clinic. Two-thirds of cases were open simple prostatectomies.

**Table 2:** Surgical Case Load, 2008-2010.

	**2008**		**2009**		**2010**		**Total**	
	
	**n**	**%**	**n**	**%**	**n**	**%**	**n**	**%**
All Cases	15	100	19	100	30	100	64	100
Simple Retropubic Prostatectomy	10	67	7	40	2	7	19	30
Inguinal Herniorrhaphy	1	7	3	15	7	23	11	17
Bilateral Palliative Orchiectomy			3	15	4	13	7	11
Unilateral Orchiectomy					3	10	3	5
Complicated Wound Debridement					3	10	3	5
I & D of Soft Tissue Mass					2	7	2	3
Orchiopexy					2	7	2	3
Breast Lumpectomy			2	10			2	3
Unilateral Hydrocelectomy					2	7	2	3
Simple Transcystic Prostatectomy			1	5			1	2
Bilateral Hydrocelectomy	1	7					1	2
Ureteropelvic Junction Repair	1	7					1	2
Open Ureteric Stone Extraction	1	7					1	2
Emergency Caesarean Section	1	7					1	2
Urethral Disruption Reconstruction			1	5			1	2
Lipoma Resection			1	5			1	2
Nephrectomy for Wilms' Tumor			1	5			1	2
Umbilical Herniorrhaphy					1	3	1	2
Ovarian Cystectomy					1	3	1	2
Suprapubic Catheter Insertion					1	3	1	2
Skin Graft to Hand					1	3	1	2
Soft Tissue Mass Resection					1	3	1	2

Percentages do not sum to 100 due to rounding

In 2009, more than 40 patients were evaluated for surgery, and a total of 20 surgical procedures were performed on 19 patients (16 male, 3 female). Nearly half of cases were simple prostatectomies (40%) with the remainder including a complex posterior urethral disruption reconstruction and a total unilateral nephrectomy for a large Wilms' tumor crossing midline.

In 2010, a total of 30 procedures were performed on 20 patients (17 male, 3 female) with over 35 cases evaluated. For the 19 cases with data available, median time per case from entering the operating room to exit was 2 hours and 14 minutes. A number of patients required multiple procedures such as one patient having serial debridements and skin grafting for a necrotizing soft-tissue infection of the hand to avoid amputation and older men requiring combined herniorrhaphy and palliative orchiectomy.

### Quality Indicators

In all three prior trips, no short-term complications were reported. No patients were returned to the operating room, and all patients were successfully discharged from the surgical team on postoperative day two. Furthermore, no surgical patients had unplanned extensions of their postoperative admission. To date, no long-term complication of any procedure performed by the surgical team has been reported.

### Student Roles

Students followed and care for patients from admission to discharge. In addition to the typical perioperative assistance provided by students in U.S. academic medical centers, students were the clinical caregivers immediately responsible for perioperative and postoperative care. Student roles noted in clinical case records included post-anesthesia monitoring, night rounding, IV line insertion, antibiotic and pain medicine administration, transporting patients, wound redressing, writing discharge summaries, obtaining consent, and counseling families. It should be emphasized that these roles were always performed with appropriate licensed medical personnel acting in a supervisory role. Due to the atomized nature of care administration at U.S. academic medical centers, it should also be noted that none of these roles are routinely available for medical students at the authors' home institution.

In the operating room, one to two students routinely assisted on each case in the typical fashion of early housestaff. Surgical skills honed included surgical approach, debridement, wound closure, and electrocauterization. Typically, each student assisted on one to two procedures each day.

## Discussion

When taken in aggregate, the data above is an example of an effort to provide top-quality surgical care to a population in dire need while also adhering to the principles of medical education embraced in U.S. academic medical centers. This demonstration project suggests that within the right supervisory environment, qualified medical students have the ability to undertake significantly more responsibility and thereby enjoy a much more substantive clinical learning experience. This model has now orchestrated three separate trips to one of the most healthcare-limited areas of the world. In every case, these trips have demonstrated the ability to care for the high-quantity, advanced surgical needs of an under-resourced area while also allowing medical students to gain critical, first-hand experience. The authors believe these clinical experiences in a foreign cultural context are what are needed to produce service-oriented future surgical leaders. These goals were satisfied while maintaining outcomes that were equal to if not better than the locally available standard of care.

Anecdotally, many healthcare professionals with international experience have questioned the authors' decision to take medical students to an under-resourced, foreign environment and expect them to function at the level of junior residents and interns. The results reported here suggest that with appropriate supervision from a thoughtfully designed multidisciplinary team, medical students can succeed in roles that are beyond the scope of "typical" medical student responsibilities. Medical students can successfully perform at the level of lower-level housestaff in the appropriate supervisory environment, and medical students are able to satisfy surgical clerkship requirements while being exposed to the opportunities for international medical service work. Since these two educational goals are widely perceived to be aspirational objectives for both graduate and postgraduate surgical training programs[2-5,15-17], the program above suggests that educators identify similar opportunities for improving the quality of medical student education, particularly in the surgical specialties. Surgical clerkships can also use these experiences to introduce service-oriented future physicians to the international opportunities available for a career in surgery.

The results above also demonstrate a gradual, but noticeable shift in types of procedures being performed from year to year. For example, the number of minor surgical procedures increased in the second and third years of the trip. Also, while two-thirds of cases in 2008 were simple open prostatectomies, only two out of the thirty total cases in 2010 were. Given that the surgical skill sets provided remained largely unchanged, one might wonder if Medishare's efforts in the area were exhausting the need for complex obstructive uropathy surgical procedures in the region.

The increased quantity of minor surgical procedures can be attributed to the dwindling surgical man-hours at *Hôpital St. Thérèse *due to overwhelming administrative responsibilities by the attending surgeon. Second, while it may be true that a backlog of chronic BPH patients had been cleared by these trips, focusing on other types of cases afforded the group opportunities to take on other rare, debilitating, and life-threatening diseases such as a Wilms' tumor or a large symptomatic abdominal mass of unknown etiology[18]. In the later years reported above, entire 20-bed wards at the hospital have been reserved for the week of the trip for patients being transferred to Hinche with "incurable" surgical needs, suggesting the region still continues to need supplemental assistance for locally available surgical services. During the years reported here, *Hôpital St. Thérèse *and peer institutions in the region had been largely unsuccessful at securing full-time coverage by even a general surgeon much less any kind of surgical subspecialist. The closest available surgical subspecialty care was in Port-au-Prince but many patients reported that these services required substantial financial resources unavailable to the typical rural Haitian citizen.

If one evaluates the case load reported above in the context of peer programs within Haiti, the number and complexity of cases performed suggests that the reported short-term surgical trips compare favorably to the productivity of other regional efforts (Table 3). Emory Medishare's first surgical trip was equivalently productive in terms of total cases performed and its later trips performed almost double the number of procedures as that performed by Partners in Health's/*Zanmi Lasante's l'Hôpital Bon Sauveur*,[19] the Central Plateau's tertiary care surgical facility also run by a combination of local Haitian and visiting international surgeons. Moreover, Emory Medishare's cases also tended to be more complex with a greater number of reconstructive versus extractive procedures, reflecting the subspecialty training of the visiting surgeons. The case load reported here also fares well against average case loads of humanitarian surgical missions worldwide with many organizations completing a similar number of cases annually that our groups' missions has completed in three weeks' time with students performing the majority of clinical care tasks [20].

**Table 3:** Comparison of case load of Emory Medishare surgical trips to year-round operation of Zanmi Lasante's surgical hospital at Cange.

	Emory Medishare (Hinche, 2008)	Emory Medishare (Hinche, 2009)	Emory Medishare (Hinche, 2010)	**Zanmi Lasante**[19] (Cange, 2002-5)
Staff Surgeons	2	2	2	2
Total Case Load	14	20	28	2167
Average Cases per Week	14	20	28	15.5

* Obstetrical cases and minor procedures omitted.

The greatest limitation of the data reported above is the reliability of follow-up data and gaps in clinical records. Although short-term complications were closely monitored by multiple team members, reporting long-term complications was dependent on local healthcare personnel. No incentives or funding were provided to our local collaborators, and all served in roles that constantly divided their attention. For example, the chief surgeon of the hospital also served as hospital director, and many of the medical staff rotated through a series of facilities on a weekly basis. Worse, intermittent communication infrastructure and natural disasters including the 2010 earthquake and the 2008 hurricanes further limited attempts to follow prior surgical patients. Given these difficulties, we believe that a good-faith effort by all parties has been made to follow-up with past patients, and the authors have been heartened by the occasional visit by a number of complex cases who recovered well and continue to live healthy, productive lives in the community. Conversely, no one has ever visited the hospital to report a poor outcome. Similarly, Project Medishare's wide network of community health workers and district supervisors - with whom members of the Emory affiliate routinely meet with - have not identified a surgical complication in the home villages of the patients served.

In addition to the specific obstacles for follow-up data, prior clinical records proved to be difficult to obtain. All record-taking at Hinche was performed with paper records that were then centrally archived. Recovering missing information such as patient demographics or anesthesia care data was prohibitively time-consuming and often times impossible due to poor records management. While this issue limited the authors' ability to report some anecdotal findings, all of the data above has been provided intact unless otherwise noted. Continuing to improve access to and breadth of clinical record-keeping is a goal for future work for tracking quality measures.

Finally, an important limitation of all short-term humanitarian missions is the potential for unethical practice of medical care that inappropriately takes advantage of a population in critical need and the implicit superiority of a well-stocked international team. The presence of relatively-inexperienced medical students can potentially further complicate the fine distinction between humanitarian relief work and conceited neocolonialism [21]. The current literature on surgical trainees practicing abroad has shown that such programs can improve trainees' clinical knowledge and cultural sensitivity[22] but can also place unacceptable risks on patient care [23]. These conflicting effects are further complicated by an educational institution's needs to balance its primary obligation to train its own students versus ethical obligations of humanitarian work (e.g., building local capacity, reducing health disparities). The consensus from previous reports is that increased institutional emphasis and ethical oversight is needed to attenuate the material and moral risks involved while maximizing potential benefit of the trainees and patients [24]. Emory Medishare's program uses an internal ethical review process previously reported by Suchdev *et *al[14] and further work is under way at the authors' home institutions to develop formal review processes across programs. The review processes consider a number of wide-ranging ethical issues such as cost-effectiveness, cultural sensitivity, local provider impact, sustainability.

## Conclusions

Short-term humanitarian surgical interventions commonly focus narrowly on bringing a "crack-team" of experienced clinicians to perform a set of highly specialized procedures for a community in need. Rarely do these activities incorporate regulated training opportunities for junior surgical trainees and even less frequently for medical students. We suggest that the model described above suggests that such interventions and medical education are not mutually exclusive. A properly designed trip model can effectively balance the surgical needs of the community with an opportunity to expose young trainees to a social and clinical experience rarely provided at their stage of medical education. Short-term surgical trips to under-resourced communities abroad have the potential to be exceptional learning opportunities and a way to expose medical students to the field of global health. Such a formative experience is the ideal opportunity to serve as an "entry point" into a longer-term experience in international humanitarian surgery. Furthermore, the evidence amassed here suggests that medical students can perform at the level of low-ranking housestaff given the opportunity and the appropriate supervision. Such opportunities are not often available in more traditional medical student curricula. Further research is needed to duplicate these results in other settings, both in the author's home country and abroad. The authors' experiences indicate that these endeavors are highly valued by all and should be expanded, but such a small program is insufficient to make a definitive recommendation. Further demonstration projects with the appropriate monitoring and evaluation are necessary to justify widespread adaptation of such an educational model. Rather than institutions entering into new international surgery ventures on their own, consortia that share opportunities among U.S. medical schools and help produce guidelines for effectively designed missions may help alleviate some of the limitations noted above [25]. In the interim, the success of the surgical trip model above suggests an acceptable level of risk to clinical care for expanding the availability of similar experiences at other U.S. teaching institutions.

## Competing interests

The authors report receiving in-kind donations of medical supplies and/or pharmaceutical products from C. R. Bard, Covidien, Medshare International, GE Healthcare, and Americares.

## Authors' contributions

IL, JM, MW, JS, MC, and VM participated in the design of the study. IL and FC supervised all data acquisition and analysis. IL and VM drafted the manuscript, while FC, MW, JM, JS, and MC contributed substantially to revisions. IL and VM take full responsibility for the integrity of the above work from inception to publication including data acquisition and analysis. All authors have read and approved the final manuscript.

## Authors' information

JM, JS, and VM have served as attending surgeons for the Emory Medishare collaborative for a collective total of six years. MW has provided non-surgical clinical support for many of the organization's activities and serves as the faculty liaison with Emory University. MC is a Haitian-born nurse trained in the U.S. and has served as Project Medishare's country director overseeing all clinical and social service operations in the *Plateau Central*. IL and FC were elective participants and trip coordinators in 2009 and 2010, respectively.
